# Retiform purpura in a patient with inflammatory bowel disease

**DOI:** 10.1016/j.jdcr.2024.08.025

**Published:** 2024-09-07

**Authors:** Turkan Banu Karatas, Nourine A.H. Kamili, Nujood Alzahrani, Taha Osman Mohammed, Jennifer Butler, Laura Johnson, Yuk Liu, Lauren Nosanov, Laura Aspey, Leslie P. Lawley

**Affiliations:** a Department of Dermatology, Emory University School of Medicine, Atlanta, Georgia; b Department of Surgery, Morehouse School of Medicine, Atlanta, Georgia; c Department of Surgery, Emory University School of Medicine, Atlanta, Georgia; d Walter L. Ingram Burn Center at Grady Memorial Hospital, Atlanta, Georgia

**Keywords:** antithrombin III deficiency, enteropathy, purpura, thrombotic vasculopathy, ulcerative colitis

## History

**Fig 1:**
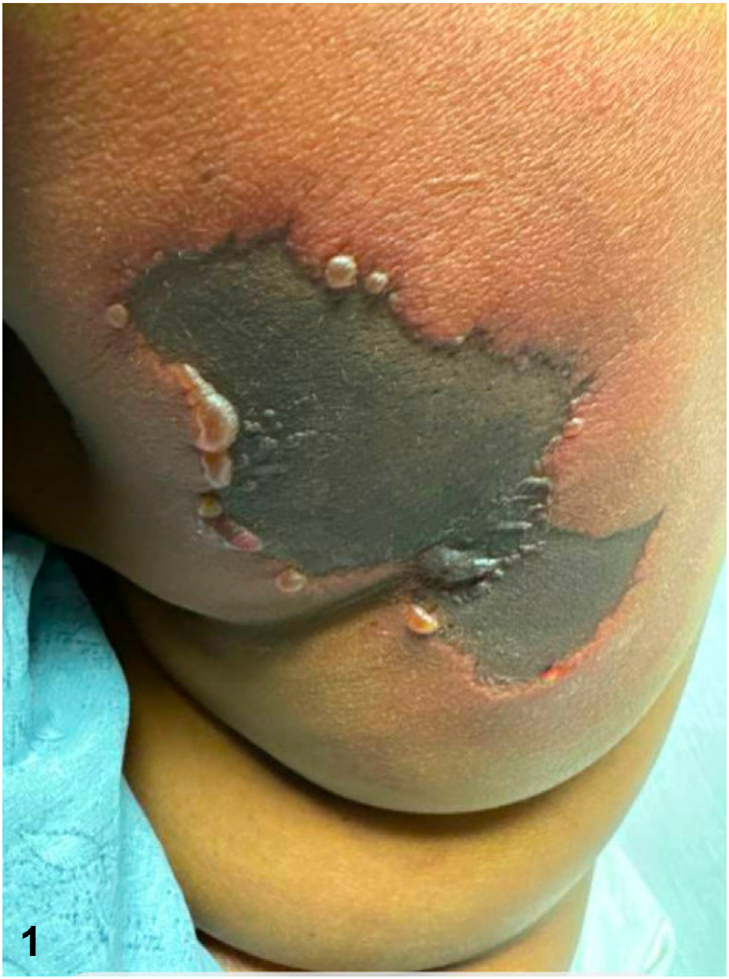

**Fig 2:**
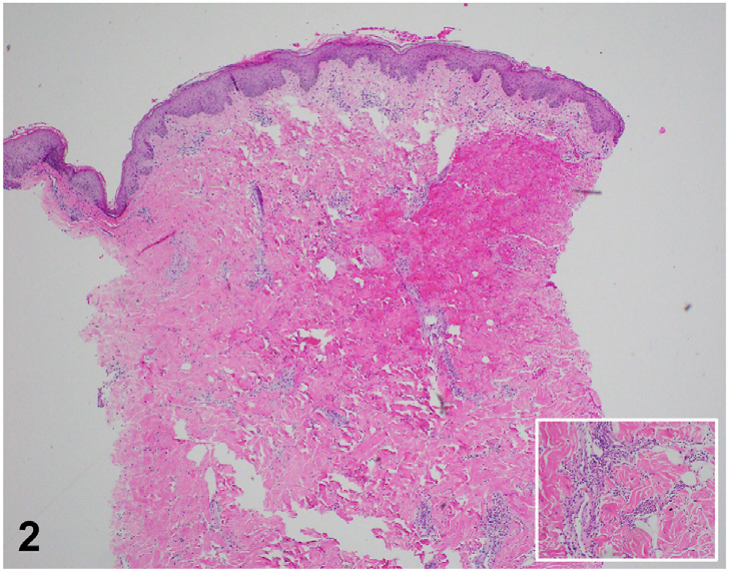

A 50-year-old woman with a history of uncontrolled ulcerative colitis (UC) on ustekinumab presents to the hospital with painful purpuric plaques on the trunk for 2 weeks. Examination revealed retiform purpuric indurated and necrotic plaques with tense hemorrhagic bullae at margins over bilateral flanks (Fig 1). Laboratory workup is notable for anemia and leukocytosis. Hypercoagulability workup showed elevated prothrombin time, partial thromboplastin time, International normalized ratio, D-dimer, and low fibrinogen. Further workup was negative for antinuclear antibody, rheumatoid factor, lupus anticoagulant, or cryoglobulins. Notably, the antithrombin III (ATIII) level was low without antithrombin antigen. Skin biopsy showed dermal hemorrhage, fibronecrotic debris, and mixed inflammatory infiltrates; no vascular calcification or vasculitis (Fig 2).

**Question 1: What is the most likely underlying diagnosis?**

A. Cutaneous thrombotic vasculopathy
B. Drug reaction to biologic
C. Pyoderma gangrenosum
D. Calciphylaxis
E. Warfarin-induced skin necrosis

**Answers:**

A. Cutaneous thrombotic vasculopathy – Correct. Patients with inflammatory bowel disease (IBD) are at risk for thromboembolism due to increased levels of coagulability factors, platelet dysfunction, and protein enteropathy leading to low antithrombin factors.^1^ Low ATIII level in this patient supports the diagnosis of cutaneous thrombotic vasculopathy secondary to ATIII deficiency in the setting of protein-losing enteropathy in uncontrolled UC. This is a rare skin manifestation of UC, and a high index of clinical suspicion is critical.^2^
B. Drug reaction to biologic – Incorrect. Ustekinumab is a biologic used in the treatment of IBD by inhibiting interleukins-23 and 12.^3^ While it can cause ecchymosis at injection sites, large purpuric and necrotic plaques are not typical. The patient’s laboratory results point to a hypercoagulability process rather than an inflammatory drug reaction.
C. Pyoderma gangrenosum – Incorrect. Pyoderma gangrenosum is a rare cutaneous manifestation of UC, presenting as rapidly progressive painful lower extremity ulcers with necrotic borders and erythema.^3^
D. Calciphylaxis – Incorrect. Calciphylaxis is a microvasculature calcification that can occur in the setting of chronic renal disease. It is less likely given preserved renal function and absent vascular calcifications on biopsy.^4^
E. Warfarin-induced skin necrosis – Incorrect. Warfarin-induced skin necrosis is a rare complication that occurs during the first few days of warfarin therapy and presents with purpuric necrotic plaques. The case patient was not on warfarin.^5^

**Question 2: Which of the following are the characteristic pathology findings for this diagnosis?**

A. Vascular calcium deposition in dermis and subcutaneous fat with ischemic necrosis of overlying epidermis and fat necrosis
B. Septal panniculitis with mixed cellular infiltrate (lymphocytes, histiocytes, giant cells, and eosinophils) and absence of vasculitis
C. Perivascular lymphocytic infiltrates with immune deposits and extravasation of erythrocytes
D. Necrotic epidermis with fibronecrotic debris and intravascular fibrin thrombi
E. Dense neutrophilic dermatosis and lymphoplasmacytic infiltrate

**Answers:**

A. Vascular calcium deposition in dermis and subcutaneous fat with ischemic necrosis of overlying epidermis and fat necrosis – Incorrect. These pathological findings can be seen in calciphylaxis.^4^
B. Septal panniculitis with mixed cellular infiltrate (lymphocytes, histiocytes, giant cells, and eosinophils) and absence of vasculitis – Incorrect. This histology can be found in erythema nodosum, another cutaneous manifestation of IBD
C. Perivascular lymphocytic infiltrates with immune deposits and extravasation of erythrocytes – Incorrect. These findings best describe lymphocytic vasculitis
D. Necrotic epidermis with fibronecrotic debris and intravascular fibrin thrombi – Correct. The characteristic histological features of thrombotic vasculopathy are necrosis and intravascular fibrin thrombi. Although the biopsy obtained from this patient did not exhibit intravascular fibrin thrombi, the overall clinical picture should always be considered and points to a diagnosis of thrombotic vasculopathy secondary to ATIII deficiency.
E. Dense neutrophilic dermatosis and lymphoplasmacytic infiltrate – Incorrect. This presentation is classically seen in pyoderma gangrenosum.^3^

**Question 3: What is the best course of management for this patient?**

A. Glucocorticoids and infliximab infusions to control UC and withhold anticoagulation so as not to exacerbate anemia
B. Infusion of ATIII to replete ATIII and prophylactic antibiotics to prevent infection
C. Glucocorticoids and infliximab infusions to control UC, and anticoagulation for thrombotic vasculopathy
D. Glucocorticoids and infliximab infusions to control UC, anticoagulation for thrombotic vasculopathy, and prophylactic antibiotics to prevent infection
E. Anticoagulation for thrombotic vasculopathy

**Answers:**

A. Glucocorticoids and infliximab infusions to control UC and withhold anticoagulation so as not to exacerbate anemia – Incorrect. Early anticoagulation is needed to prevent progressive cutaneous infarction.^2^
B. Infusion of ATIII to replete ATIII and prophylactic antibiotics to prevent infection – Incorrect. ATIII repletion alone is insufficient anticoagulation to prevent progressive cutaneous infarction. Prophylactic antibiotics are generally not needed.
C. Glucocorticoids and infliximab infusions to control UC, and anticoagulation for thrombotic vasculopathy – Correct. Early anticoagulation is needed to prevent progressive cutaneous infarction and anti-inflammatory control of UC flare-up is needed to prevent further enteric loss of ATIII and decrease hypercoagulable state.^1^ The presented patient was started on therapeutic anticoagulation with argatroban and on high-dose glucocorticoids and infliximab.
D. Glucocorticoids and infliximab infusions to control UC, anticoagulation for thrombotic vasculopathy, and prophylactic antibiotics to prevent infection – Incorrect. Prophylactic antibiotics in this setting are not needed and should be avoided for appropriate antibiotic stewardship. Antibiotics should only be used if cutaneous lesions appear infected.
E. Anticoagulation for thrombotic vasculopathy – Incorrect. While early anticoagulation is needed to prevent progressive infarction, active UC creates a hypercoagulable state that should be addressed with UC control.

## Conflicts of interest

None disclosed.

## References

[1] Komatsu Y.C., Capareli G.C., Boin M.F., Lellis R., Freitas T.H., Simone K. (2014). Skin gangrene as an extraintestinal manifestation of inflammatory bowel disease. An Bras Dermatol.

[2] Cheeley J., Morales-Pico B., John S. (2018). Cutaneous thrombotic vasculopathy related to poorly controlled ulcerative colitis. JAAD Case Rep.

[3] Miklusiak K., Miklusiak K., Kaczmarczyk O., Cibor D., Zwolińska-Wcisło M. (2023). Ustekinumab in the treatment of acute disseminated pyoderma gangrenosum in a patient with Crohn's disease. Dermatol Reports.

[4] Gallo Marin B., Aghagoli G., Hu S.L., Massoud C.M., Robinson-Bostom L. (2023). Calciphylaxis and kidney disease: a review. Am J Kidney Dis.

[5] Nazarian R.M., Van Cott E.M., Zembowicz A., Duncan L.M. (2009). Warfarin-induced skin necrosis. J Am Acad Dermatol.

